# Multimorbidity and Depression Incidence Among Middle-Aged and Older Adults in China

**DOI:** 10.1093/geroni/igaa057.823

**Published:** 2020-12-16

**Authors:** Fei Tang, Elizabeth Vasquez

**Affiliations:** 1 University at Albany (SUNY), Albany, New York, United States; 2 University at Albany (SUNY), Rensselaer, New York, United States

## Abstract

Previous studies suggested that multimorbidity (co-occurrence of two or more chronic conditions) is associated with increased risk of depression. However, limited studies have examined the relationships between multimorbidity and depression among Chinese middle-aged and older adults. The current study aimed to evaluate the associations between multimorbidity and incidence of depression and the potential mediation effect of functional limitation among Chinese middle–aged and older adults. Data of 8,093 individuals who participated in both wave 1 (2011) and wave 4 (2015) of China Health and Retirement Longitudinal Study (CHARLS) and were free of depression in wave 1 were included in the study. Multiple log-binomial regression models were used to evaluate the associations between multimorbidity and incident depression. Mediation analysis was conducted to examine the effect of functional limitation. A third of participants in our study were identified as having multimorbidity in wave 1 (N=2,479) and 23% participants were free of depression in wave 1 but had depression in wave 4 (N=1,827). After adjusting for potential confounders, multimorbidity was observed to be associated with depression incidence (RR: 1.31; 95% CI: 1.21 – 1.42). In addition, functional limitation mediated the relationship between multimorbidity and depression incidence. Our findings add to the literature on the potential associations between multimorbidity and depression incidence among Chinese middle-aged and older adults. Furthermore, the relationship between multimorbidity and depression incidence was observed to be mediated by functional limitation. Interventions that improve functional ability among Chinese middle-aged and older adults could potentially attenuate the effect of multimorbidity on depression incidence.

